# Efficient Generation of Neural Stem Cells from Embryonic Stem Cells Using a Three-Dimensional Differentiation System

**DOI:** 10.3390/ijms22158322

**Published:** 2021-08-03

**Authors:** Sang-Hoon Yoon, Mi-Rae Bae, Hyeonwoo La, Hyuk Song, Kwonho Hong, Jeong-Tae Do

**Affiliations:** Department of Stem Cell and Regenerative Biotechnology, Konkuk Institute of Technology, Konkuk University, 120 Neungdong-ro, Gwangjin-gu, Seoul 05029, Korea; kei99137@naver.com (S.-H.Y.); mirae9607@naver.com (M.-R.B.); lahw94@gmail.com (H.L.); songh@konkuk.ac.kr (H.S.); hongk@konkuk.ac.kr (K.H.)

**Keywords:** embryonic stem cells, neural differentiation, 3D culture

## Abstract

Mouse embryonic stem cells (ESCs) are useful tools for studying early embryonic development and tissue formation in mammals. Since neural lineage differentiation is a major subject of organogenesis, the development of efficient techniques to induce neural stem cells (NSCs) from pluripotent stem cells must be preceded. However, the currently available NSC differentiation methods are complicated and time consuming. This study aimed to propose an efficient method for the derivation of NSCs from mouse ESCs; early neural lineage commitment was achieved using a three-dimensional (3D) culture system, followed by a two-dimensional (2D) NSC derivation. To select early neural lineage cell types during differentiation, *Sox1*-GFP transgenic ESCs were used. They were differentiated into early neural lineage, forming spherical aggregates, which were subsequently picked for the establishment of 2D NSCs. The latter showed a morphology similar to that of brain-derived NSCs and expressed NSC markers, Musashi, Nestin, N-cadherin, and Sox2. Moreover, the NSCs could differentiate into three subtypes of neural lineages, neurons, astrocytes, and oligodendrocytes. The results together suggested that ESCs could efficiently differentiate into tripotent NSCs through specification in 3D culture (for approximately 10 days) followed by 2D culture (for seven days).

## 1. Introduction

Pluripotent stem cells (PSCs) are valuable tools for the study of differentiation and tissue development since they can differentiate into various cell types in the body. Directed differentiation into specialized cell types is also a stratagem for obtaining a pure population of cells of a certain tissue. Neural lineage differentiation from PSCs is especially important because, unlike other tissue cells, neural cells are not available from living organisms. The establishment of an efficient protocol for the differentiation of PSCs into specific somatic lineage is an important step in stem cell therapy [1,2,3,4]. Many researchers have developed the appropriate technology for the differentiation of embryonic stem cells (ESCs) into neurons or neural stem cells (NSCs). Initially, the protocol for neuronal differentiation from PSCs was based on suspension culture in the presence of retinoic acid (RA). Co-culture with stromal cells facilitated neural differentiation from ESCs due to stromal cell-derived inducing activity in the co-culture system [5,6]. Using human PSCs, Chambers et al. had demonstrated that blocking BMP and TGFβ signaling pathways (also called dual SMAD inhibition) by Noggin and SB431542 (a SMAD signaling inhibitor) could rapidly cause human ESCs to differentiate into early neuroectoderm, followed by the formation of neural rosettes [7].

Recently, we developed an in vivo differentiation method, by which ESCs could efficiently differentiate into NSCs in vivo or in teratoma [8]. *Olig2*-GFP-expressing cells formed in teratoma were sorted and established as NSCs in vitro. The method was also effective in partially reprogrammed cells that lacked embryoid body formation and in vitro differentiation abilities [9]. In fact, the method can be a universal protocol as well, except for the fact that it is time-consuming and slightly complicated; teratoma formation takes 4–6 weeks, and additional steps for the selection and proliferation of early neural lineage cells are required [8]. This study aimed to develop a simpler method to generate NSCs from ESCs via a three-dimensional (3D) extracellular matrix gel, the Matrigel system, which shortens the time to establish stable NSC lines. The method did not require the addition of neural induction factors to induce neural differentiation, since the latter is a kind of primary direction of lineage specification during gastrulation. The established NSCs showed typical characteristics of NSCs, including morphology, differentiation ability, and gene expression profiles.

## 2. Results

### 2.1. Early Neural Lineage Commitment by 3D Culture System

Pluripotent stem cells (PSCs) can differentiate into any cell type belonging to any body tissue. The directed differentiation of PSCs and obtaining a pure population of preferred cells are almost impossible, since various types of unwanted cells are induced along with the wanted cells and form a heterogeneous cell population as a result. Thus, lineage-specific markers have been used for the efficient selection of wanted cells only. To select early neural lineage cell types during the differentiation of PSCs, we used transgenic ESCs, in which green fluorescence protein (GFP) was expressed under the control of *Sry-related-HMG box 1* (*Sox1*), called *Sox1*-GFP [10]. *Sox1* plays a major role in neural specification during the early formation of neuroepithelial cells in the neural tube [10,11,12]. *Sox1* is also important for neural development and differentiation in both humans and mice in vitro [13]. Thus, we hypothesized that the expression of Sox1 during the differentiation of PSCs can identify early stages of neural development, thereby helping the optimization of differentiation protocols. In other words, the *Sox1*-GFP marker could possibly identify differentiation into early neural lineage efficiently.

**Figure 1:**
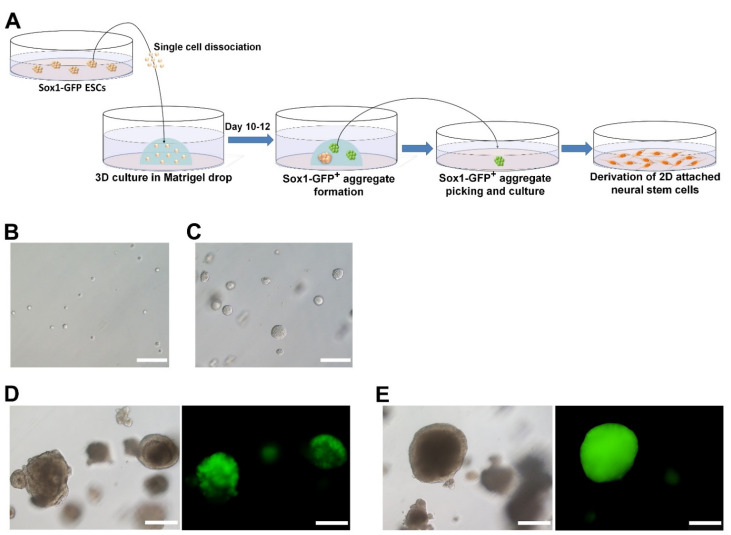
Differentiation of *Sox1*-GFP ESCs into neural stem cells (NSC). (**A**) Schematic illustration of the NSC induction protocol. (**B**) Dissociated single cells of *Sox1*-GFP ESCs embedded in Matrigel drops. (**C**) Aggregate formation of *Sox1*-GFP ESCs in Matrigel on day three. (**D**) *Sox1*-GFP-expressing cells appeared on day seven after culture in the Matrigel drop. (**E**) On day 10, *Sox1*-GFP-expressing aggregates were picked and used for NSC derivation. Scale bars = 200 µm.

**Figure 2:**
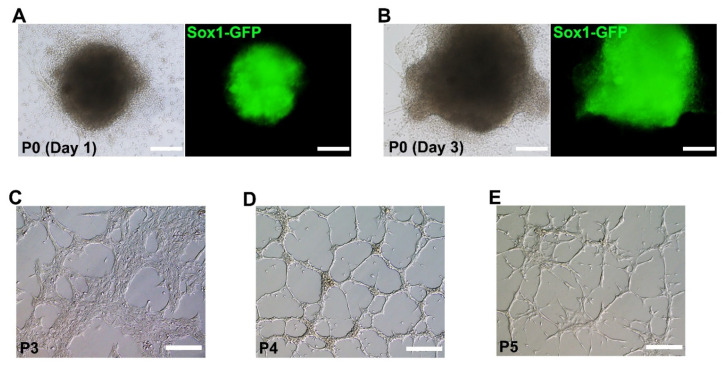
Establishment of neural stem cells (NSCs) using *Sox1*-GFP-expressing aggregates. GFP-expressing aggregates attached onto a gelatin-coated dish on day one (**A**) and day three (**B**) after transferring to a new dish. (**C**) NSCs established from aggregates at passages (P) 3, 4, and 5 showed typical NSC morphology (**C**,**D**, and **E**, respectively). Scale bars = 200 µm.

To induce differentiation, undifferentiated *Sox1*-GFP ESCs were dissociated, seeded on the Matrigel drop (3000 cells/50 μL), and cultured in Epi medium (Figure 1A). Epi medium is a conventional medium for the culture of epiblast stem cells (EpiSCs). Here, Epi medium was used to induce the differentiation of ESCs in Matrigel drops (Figure 1B). In this 3D culture system, *Sox1*-GFP ESCs proliferated and formed spherical aggregates, a kind of ESC colony in a 3D environment, on approximately day three of the culture (Figure 1C). However, some of the *Sox1*-GFP^+^ aggregates (approximately 30% of total) began to form two-layered aggregates (Figure 1D), which started expressing *Sox1*-GFP (*Sox1*-GFP^+^) on day seven after culture in the Matrigel drop. On day 10, aggregates composed of cells homogeneously expressing *Sox1*-GFP appeared (Figure 1E), and these were subsequently used for NSC derivation (Figure 2A). The results indicated that *Sox1*-GFP ESCs could be differentiated into an early neural lineage, or neuroepithelial cells, in seven days under a 3D environment.

### 2.2. Establishment of NSCs Using Sox1-GFP-Expressing Aggregates

We removed the Matrigel using Cell Recovery Solution and isolated the GFP-expressing aggregates. On day 10, aggregates in which almost all cells were expressing *Sox1*-GFP were picked and transferred to gelatin-coated dishes with NSC expansion medium containing bFGF and EGF, where cells were cultured in 2D (Figure 2A). GFP-expressing aggregates were attached onto the dish, and formed outgrowths (Figure 2A,B). Aggregates were then dissociated into single cells and transferred onto gelatin-coated dishes, where bipolar cells (typical NSC morphology) were formed (Figure 2C). Actively self-renewing NSCs were established through subsequent culture (Figure 2D,E). The results suggested that ESCs efficiently differentiated into 2D NSCs within 17 days through specification in 3D culture (for approximately 10 days) followed by 2D culture (for seven days).

### 2.3. Characterization of Established NSCs Using NSC Markers

**Figure 3:**
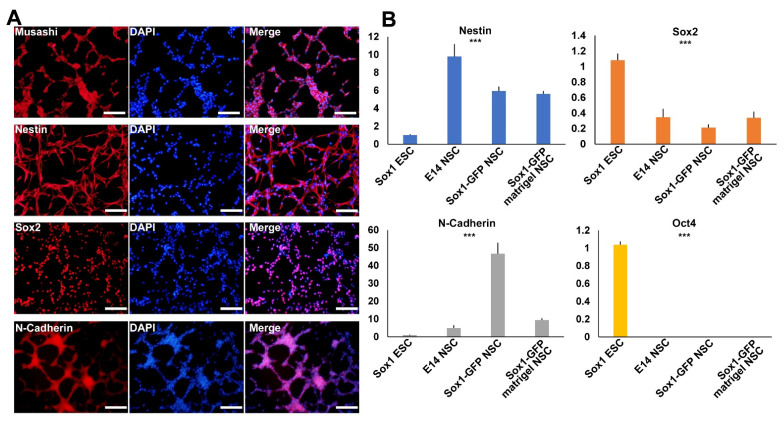
Characterization of the established NSCs. (**A**) Immunofluorescence analysis of established NSCs using NSC-specific markers, namely Musashi, Nestin, Sox2, and N-cadherin. Scale bars = 200 µm. (**B**) Real-time RT-PCR analysis of established NSCs using NSC marker genes, namely Nestin, Sox2, N-cadherin, and pluripotent marker gene Oct4. One-way ANOVA: *** *p* < 0.001 (*n* = 3).

To check whether the established NSCs showed typical NSC characteristics, we conducted immunocytochemistry and real-time RT-PCR analyses using major NSC markers. NSC-specific expression was confirmed using antibodies for Musashi, Nestin, Sox2, and N-cadherin (Figure 3A). Four NSC markers were expressed in a representative NSC line. Real-time RT-PCR analysis also confirmed that the established NSCs expressed NSC markers, such as *Nestin*, *Sox2*, and *N-cadherin*, but not the pluripotency marker, *Oct4* (Figure 3B). *Sox2*, a marker for both pluripotent stem cells and NSCs, was highly expressed in both ESCs and NSCs. The results confirmed that NSCs established through lineage specialization in 3D and 2D showed typical NSC marker expression.

### 2.4. Transcriptomic Comparison of NSCs and ESCs

**Figure 4:**
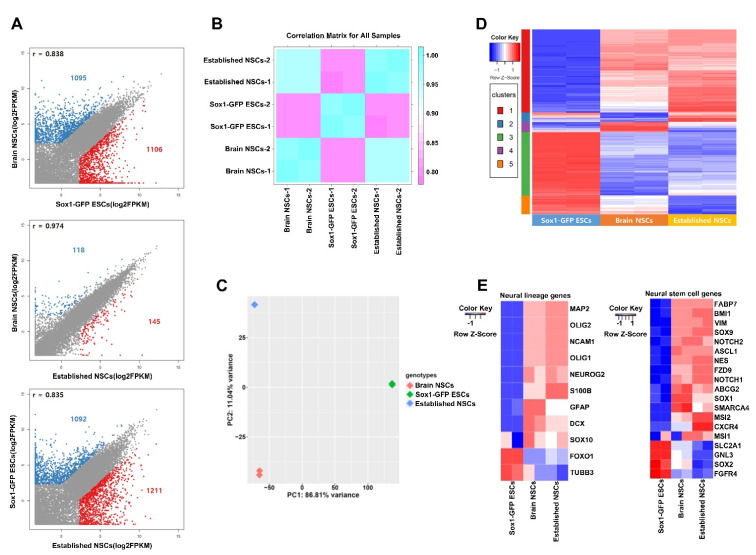
Transcriptome analysis of three cell lines: established NSCs, brain NSCs, and *Sox1*-GFP ESCs. (**A**) Scatterplot analysis of three cell lines. (**B**) Correlation matrix analysis of three cell lines. (**C**) Principle component analysis (PCA) of bulk RNA-seq data from three cell lines. (**D**) Heatmap clusters of DEGs between three cell lines. (**E**) Heatmap of lineage-specific gene expression in neural cells and neural stem cells.

We performed RNA-seq analysis of the established NSCs and compared their transcriptome to brain-derived NSCs and *Sox1*-GFP ESCs. Our analysis revealed 2201, 263, and 2303 genes to be differentially expressed in brain-derived NSCs vs. *Sox1*-GFP ESCs, brain-derived NSCs vs. established NSCs, and *Sox1*-GFP ESCs vs. established NSCs, respectively (FPKM > 5, fold change > 5) (Figure 4A). The correlation matrix showed that the gene expression pattern in established NSCs was closer to that in brain-derived NSCs than that in *Sox1*-GFP ESCs (Figure 4B). Principle component analysis revealed that the established NSCs were closer to brain-derived NSCs on PC1 axes (Figure 4C). Although there was a difference on the PC2 axis, it was only 11.04% between the two cell lines (Figure 4C). A heatmap revealed the genes expressed in established NSCs and brain-derived NSCs to largely overlap each other. Hierarchical clustering analysis using the differentially expressed genes (DEGs) between different cell types revealed five clusters showing a unique signature: ESC-specific genes downregulated (cluster 1) and upregulated (cluster 3), brain-derived NSC-specific genes downregulated (cluster 2) and upregulated (cluster 4), and genes with different expression patterns across all three samples (cluster 5) (Figure 4D). According to cluster 1, which showed the greatest commonality between established NSCs and brain-derived NSCs, the upregulation of genes related to small GTPase-mediated signal transduction, nervous system development, and axonogenesis was indicated (Supplementary Figure S1). Voss et al. also reported that GTPase is associated with neural stem cells [14]. However, there was a difference between the two NSC lines. Cluster 2 showed differences in the expression of genes related to immunity and apoptosis, and cluster 4 showed differences in the expression of genes related to steroids. The genes in clusters 2 and 4, however, were not significantly associated with neural lineage or NSC signatures. Thus, we further analyzed the genes specifically associated with neural lineage or NSC signatures (Figure 4E). The expression levels of neural lineage- and NSC-related genes were similar between the established NSCs and brain-derived NSCs, confirming that the molecular signatures of newly established NSCs were similar to that of brain-derived NSCs.

### 2.5. Differentiation of the Established NSCs into Neurons and Glial Cells

**Figure 5:**
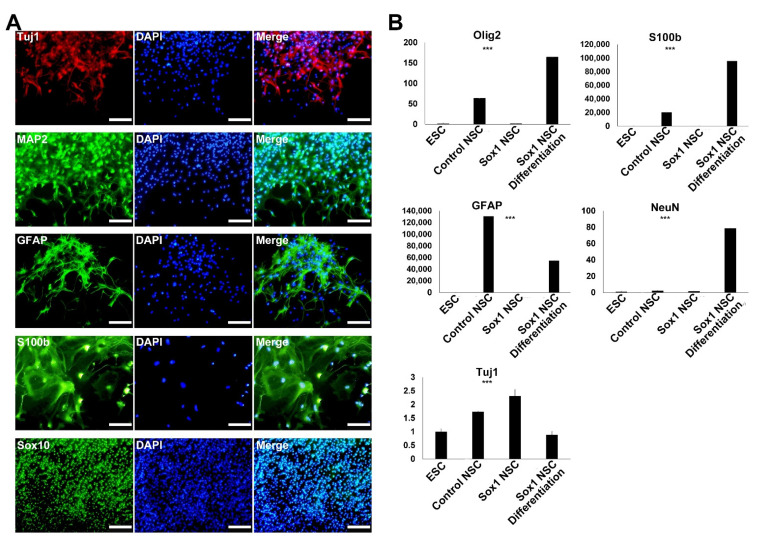
Differentiation potential of the established NSCs. (**A**) Immunofluorescence analysis of cells differentiated from NSCs using neural subtype markers, including neurons (Tuj1 and MAP2), astrocytes (GFAP and S100b), and oligodendrocytes (Sox10^+^). (**B**) Real-time RT-PCR analysis confirmed the differentiation potential of established NSCs into neuronal cells (*Tuj1* and *NeuN*), astrocytes (*GFAP* and *S100b*), and oligodendrocytes (*Olig2*). One-way ANOVA: *** *p* < 0.001 (*n* = 3).

NSCs are multipotent stem cells that can differentiate into various subtypes of neurons and glial cells. Thus, validating the ability of established NSCs to differentiate into neurons, astrocytes, and oligodendrocytes is important. We confirmed the same by immunocytochemistry and real-time RT-PCR analysis (Figure 5). NSCs were randomly differentiated into neurons and glial cells in neuronal differentiation medium. Immunocytochemistry revealed that the established NSCs could differentiate into neurons (Tuj1^+^ and MAP2^+^), astrocytes (GFAP^+^ and S100b^+^), and oligodendrocytes (Sox10^+^) (Figure 5A). S100b being a late astrocyte marker, the finding indicated that *Sox1*-GFP ESC-derived NSCs could differentiate into the late astrocyte stage. Quantitative RT-PCR analysis also confirmed the expression of neuronal (*Tuj1* and *NeuN*), astrocyte (*GFAP* and *S100b*), and oligodendrocyte (*Olig2*) markers, suggesting the tri-potent differentiation ability of newly derived NSCs (Figure 5B).

## 3. Discussion

In this study, we developed an efficient method for the differentiation of PSCs into tripotential NSCs through specification in 3D culture followed by 2D adhesion culture, which takes less than three weeks in total. The established NSCs could express NSC-specific markers and differentiate into neurons, astrocytes, and oligodendrocytes, similar to typical NSCs. To provide a 3D environment, we used Matrigel, an extracellular matrix (ECM) that forms gel at 37 °C. Matrigel-embedded ESCs in Epi medium, without neural induction conditions, such as RA treatment, SMAD inhibition, and a co-culture system, could differentiate into *Sox1*-expressing early neural lineage cells. This may be because neural lineage commitment is one of the earliest differentiation processes, and is a kind of default event in mouse embryonic development [10]. Naïve pluripotent mouse ESCs may not remain in a naïve state in primed PSC medium (Epi medium), and are rather committed to the cell types that occur first in the developmental stage, also referred to as neural commitment. Another possible mechanism is that Matrigel provides a differentiation-friendly environment for PSCs. Previous reports had suggested that many cell lines are prone to differentiation, rather than proliferation, in the 3D matrix environment [15,16]. Thus, our differentiation method is suitable for NSC derivation, though not for tissue cells that arise later during embryonic development.

We used *Sox1*-GFP ESCs to monitor the early neural lineage commitment. *Sox1*-GFP, which is a very early neuroectoderm marker, is transiently expressed in early neuroectoderm cells and becomes inactive during further differentiation both in vivo and in vitro [10]. Accordingly, the established adherent NSCs were *Sox1*-GFP-negative since they had already passed the neuroectoderm stage. In our previous reports, we had used *Olig2*-GFP ESCs to derive NSCs through teratoma formation [8]. Since *Olig2* is known to be expressed in more specialized cells, like radial-glial cells, NSCs, and oligodendrocyte progenitors, and we needed a marker for the earlier stage of neural lineage, *Olig2*-GFP did not fit our experimental purpose. Using *Sox1*-GFP ESCs, we shortened the selection of neural lineage cell types and found a novel and efficient method to generate NSCs from mouse PSCs.

Previously, we had developed many differentiation methods to derive NSCs from mouse ESCs. NSC differentiation can be induced in vivo, either in teratoma or in brain tissues [8,17]. Teratoma was generated in the body of an immunodeficient mouse after injection of *Olig2*-GFP ESCs. *Olig2*-GFP-positive cells could be isolated from the teratoma and cultured for NSC establishment. ESC-derived NSCs could be established as well by culturing the brain tissue formed in chimeric embryos. However, the methods have the limitations of being time-consuming and complicated, since many steps are required, including the specification in the mouse body and isolation step, prior to the establishment of NSCs. To compensate for these drawbacks, in this study, we have excogitated a simpler way to imitate the in vivo environment by providing the 3D culture environment in vitro.

In regenerative medicine, damaged cells should be restored for proper function or replaced by transplanted healthy cells [18]. Since NSCs form an attractive cell type for neural regeneration, the development of appropriate protocols to differentiate PSCs into NSCs is important. When NSCs are transplanted into a damaged nervous system, they will repopulate the niche through self-renewal, differentiate into postmitotic mature cell types, including neurons and glial cells, in response to intracellular signals, and eventually replace the damaged nervous tissues. Our novel method can be applied to human PSCs, and NSCs differentiated from human PSCs can be used to treat a variety of neurodegenerative diseases. However, current stem cell therapy technology is limited by transplantation efficiency since the transplanted cells cannot survive in the long-term and are significantly reduced in the in vivo environment. Thus, for our NSC derivation method to be applied to the clinic, optimization of the transplantation technique must be conducted first. Moreover, the development of ECM material to substitute Matrigel, which is not approved by the DFA for clinical purposes, is also imperative.

## 4. Materials and Methods

### 4.1. Culture of Sox1-GFP ESCs

In this study, *Sox1*-GFP mouse ESCs containing GFP transgene under the control of the *Sox1* promoter were used [1,19]. These ESCs were derived from the 129P2/Ola strain. They were incubated at 37 °C in a 5% CO_2_ atmosphere under feeder-independent conditions. Single cell passages were performed at 2–3-day intervals using trypsin-EDTA (Gibco) in a gelatin-coated dish. The medium used was serum-free N2B27 medium (DMEM/F12 medium (Gibco), neurobasal medium (Gibco) 1:1 mixture, N2 supplement (Gibco), B27 supplement (Gibco), 100× penicillin/streptomycin/glutamine (Gibco), leukemia inhibitory factor (LIF, ESGRO; Chemicon), 3 μM of GSK inhibitor (Sigma), and 1 μM of MEK inhibitor (Sigma)).

### 4.2. Differentiation via the 3D Matrigel Culture System

The day before, Matrigel was placed in 4 °C and prepared in a liquid state. The ESCs were made into a single-cell preparation using trypsin-EDTA (Gibco). The cell number was counted using a hematocytometer, and 3000 single cells were seeded into 50 μL of Matrigel (Corning), in drop form, in a four-well dish. The Matrigel drop was allowed to solidify for an hour, incubated at 37 °C in a 5% CO_2_ atmosphere. To the solidified Matrigel drop in the four-well dish, Epi medium was added. The cells were incubated in Epi medium, which consisted of DMEM/F12 (Gibco), 100× penicillin/streptomycin/glutamine (Gibco), 5% knockout serum (Gibco), 100× non-essential amino acid (NEAA, Gibco), 20 ng/mL Activin A supplement (PeproTech), and 10 ng/mL bFGF supplement (R&D system), every three days. To form neurospheres, cells were cultured in Epi medium for 10–12 days.

### 4.3. Derivation and Expansion of Neural Stem Cells (NSCs)

After incubating the *Sox1*-GFP ESCs in Matrigel, the latter was removed using Cell Recovery Solution (Corning) on day 11–12. Using a fluorescence microscope, only colonies expressing GFP and resembling the morphology of neurospheres were picked and transferred to a 24-well gelatin-coated dish with NSC expansion medium (DMEM/F12 (Gibco), 100× penicillin/streptomycin/glutamine (Gibco), B27 supplement (Gibco), 10 ng/mL of epidermal growth factor (EGF, Gibco), and 10 ng/mL of basic fibroblast growth factor (bFGF, R&D system)) and cultured for 3–4 days. The expanded neurospheres were dissociated into single cells by a 0.25% trypsin (Gibco) treatment and passaged every 2–3 days in NSC expansion medium.

### 4.4. Differentiation of NSCs into Neurons and Glial Cells

NSCs were differentiated into neurons and glial cells after three weeks of culture in neuronal differentiation medium (1:1 mixture of DMEM/F12 medium (Gibco) and neurobasal medium (Gibco) supplemented with 1% fetal bovine serum (Hyclone), N2 (Gibco) and B27 supplements (Gibco), and 1× penicillin/streptomycin/glutamine (Gibco)).

### 4.5. Immunocytochemistry (ICC)

The established NSCs were stained using antibodies against Nestin, Sox2, Pax6, and Musashi, which are markers of NSCs. For immunocytochemistry, cells were fixed with 4% paraformaldehyde (PFA) at 4 °C for 20 min. After washing with phosphate-buffered saline (PBS, Welgene), they were incubated in PBS containing 3% bovine serum albumin (BSA, Gibco) and 0.3% Triton X-100 (Sigma) at room temperature (24 °C, RT) for 45 min. The primary antibody used was anti-Nestin (Nestin; monoclonal, 1:500, Millipore), anti-Sox2 (Sox2; polyclonal, 1:500, Millipore), anti-Musashi-1 (Musashi-1; polyclonal, 1:500, Millipore), and anti-Pax6 (Pax6; monoclonal, 1:500, Abcam). For verification, a secondary antibody labeled with a fluorescent material (Alexa Fluor 488 or 568; molecular probes, Eugene, OR, USA) was used according to the manufacturer’s instructions. DAPI is a fluorescent dye that binds to the adenine-thymine-rich region of DNA, and is a marker that indicates the position of the nucleus by nuclear staining. DAPI was diluted 1000:1 in wash buffer (0.3% Triton-X 100, Sigma), and DAPI staining was performed to confirm the location of the nucleus.

### 4.6. RNA Isolation and Real-Time PCR Analysis

RNA extraction was performed using TRIzol reagent (Invitrogen) according to standard protocols; cDNA was synthesized from 1 mg of total RNA using SuperScript III reverse transcriptase (Invitrogen, Grand Island, NY, USA). Real-time polymerase chain reaction (real-time PCR) was performed in triplicate using TOPreal™ qPCR 2x PreMIX (Enzynomics) on a Roche LightCycler 480. Thermal cycling followed the program of 45 cycles of 10 s at 95 °C, 10 s at 60 °C, and 20 s at 72 °C. The primers for real-time PCR are shown in Table 1. We corrected for differences in PCR efficiency between the target and reference loci using efficiency correction in the Relative Quantification Software (Roche LC 480, Roche, Basel, Switzerland).

### 4.7. RNA Sequencing

Total RNA was isolated using TRIzol (Thermo Fisher Scientific, 15596026), and the RNA samples were converted into cDNA using the TruSeq Stranded mRNA Sample Prep Kit (Illumina). DNA contamination was removed using DNase. Polyadenylated RNA was selected and purified using oligo dT-conjugated magnetic beads. The mRNA was physically fragmented, and the fragmented mRNA was converted into single-stranded cDNA using reverse transcriptase and random hexamer primers. Actinomycin D was added to inhibit DNA-dependent synthesis of the second strand. Double-stranded cDNA was generated by removing the RNA template and synthesizing the second strand in the presence of dUTP (deoxy-ribo-uridine triphosphate) instead of dTTP (deoxythymidine triphosphate). A single A base was added to the 3′ end to facilitate ligation of sequencing adapters containing a single T base overhang. Adapter-ligated cDNA was amplified by a polymerase chain reaction to increase the size of the sequence-ready library. During amplification, the polymerase stopped when it encountered a U base, hence rendering the second strand a poor template. Accordingly, the amplified material used the first strand as a template, thereby preserving the strand information. The final cDNA libraries were analyzed for size distribution using an Agilent Bioanalyzer (DNA 1000 kit; Agilent), quantitated by qPCR (Kapa Library Quant Kit; Kapa Biosystems, Wilmington, MA, USA), and then sequenced.

### 4.8. Sequence Read Processing

The sequence quality was examined using FastQC (v0.11.5) and the files were determined to be eligible for further analysis. Quality assessed reads were aligned to a UCSC mouse mm10 genome assembly using a STAR (v2.5.2b) aligner; STAR results were used to calculate the average expression of each gene in FPKM, and the acquired values were used for drawing scatter plots in R (v3.6.1). For heatmaps, FPKM values of individual samples and the heatmap2 function of R’s Gplots (v3.0.1.1) package were used. DEGs were determined by using previously mentioned values as the threshold. In all cases, DEGs were identified with aforementioned cutoff values using calculations with R. With the resulting DEGs from the scatter plot and heatmap, gene ontology and KEGG pathway analyses were performed using DAVID (v6.8).

### 4.9. Statisitical Analysis

All experiments were performed in triplicate and reproduced as independent samples. Data are presented as means ± standard deviations of the mean (SD). Differences were assessed using one-way analysis of variance (ANOVA) with Tukey honestly significant difference (HSD) post hoc for multiple comparisons appropriately; differences with *p*-values of less than 0.05 were considered statistically significant.

## Figures and Tables

**Table 1:** Primer sets used for real-time PCR.

	Forward	Reverse
*Nestin*	5′-AAGTTCCCAGGCTTCTCTTG-3′	5′-GTCTCAAGGGTATTAGGCAAGG-3′
*Sox2*	5′-GAACGCCTTCATGGTATGGTCC-3′	5′-CGGGTGCTCCTTCATGTGC-3′
*N-cadherin*	5′-GTACCGCAGCATTCCATTCAGG-3′	5′-GGCAATCAAGTGGAGAACCCC-3′
*Oct4*	5′-GGCTTCAGACTTCGCCTTCT-3′	5′-TGGAAGCTTAGCCAGGTTCG-3′
*Olig2*	5′-GCCTGCTCAAGTCACCGTCG-3′	5′-CACACGCCATCAACACTGGTCGC-3′
*S100b*	5′-GGTTGCCCTCATTGATGTCTTCC-3′	5′-CATCCCCATCTTCGTCCAGCC-3′
*GFAP*	5′-CTGATGTCTACCAGGCGGAGC-3′	5′-CCAGGTTGTTCTCTGCCTCCAG-3′
*NeuN*		
*Tuj1*	5′-GCTCACGCAGCAGATGTTCG-3′	5′-GGATGTCACACACGGCTACC-3′
*Actb*	5′-CGCCATGGATGACGATATCG-3′	5′-CGAAGCCGGCTTTGCACATG-3′

## Data Availability

The data that support the findings of this study are available from the corresponding author upon reasonable request.

## Supplementary Materials

The following are available online at https://www.mdpi.com/article/10.3390/ijms22158322/s1.

## References

[1] Ying Q.-L., Smith A.G. (2003). Defined conditions for neural commitment and differentiation. Methods Enzymol..

[2] Conti L., Pollard S.M., Gorba T., Reitano E., Toselli M., Biella G., Sun Y., Sanzone S., Ying Q.-L., Cattaneo E. (2005). Niche-independent symmetrical self-renewal of a mammalian tissue stem cell. PLoS Biol..

[3] Takebe T., Sekine K., Enomura M., Koike H., Kimura M., Ogaeri T., Zhang R.-R., Ueno Y., Zheng Y.-W., Koike N. (2013). Vascularized and functional human liver from an iPSC-derived organ bud transplant. Nature.

[4] Choi H.W., Kim J.S., Choi S., Hong Y.J., Kim M.J., Seo H.G., Do J.T. (2014). Neural stem cells differentiated from iPS cells spontaneously regain pluripotency. Stem Cells.

[5] Bain G., Kitchens D., Yao M., Huettner J.E., Gottlieb D.I. (1995). Embryonic stem cells express neuronal properties in vitro. Dev. Biol..

[6] Kawasaki H., Mizuseki K., Nishikawa S., Kaneko S., Kuwana Y., Nakanishi S., Nishikawa S.-I., Sasai Y. (2000). Induction of midbrain dopaminergic neurons from ES cells by stromal cell–derived inducing activity. Neuron.

[7] Chambers S.M., Fasano C.A., Papapetrou E.P., Tomishima M., Sadelain M., Studer L. (2009). Highly efficient neural conversion of human ES and iPS cells by dual inhibition of SMAD signaling. Nat. Biotechnol..

[8] Hong Y.J., Kim J.S., Choi H.W., Song H., Park C., Do J.T. (2016). In vivo generation of neural stem cells through teratoma formation. Stem Cells Dev..

[9] Kim J.S., Hong Y.J., Choi H.W., Song H., Byun S.J., Do J.T. (2017). Generation of in vivo neural stem cells using partially reprogrammed cells defective in in vitro differentiation potential. Oncotarget.

[10] Aubert J., Stavridis M.P., Tweedie S., O’Reilly M., Vierlinger K., Li M., Ghazal P., Pratt T., Mason J.O., Roy D. (2003). Screening for mammalian neural genes via fluorescence-activated cell sorter purification of neural precursors from Sox1-gfp knock-in mice. Proc. Natl. Acad. Sci. USA.

[11] Wood H.B., Episkopou V. (1999). Comparative expression of the mouse Sox1, Sox2 and Sox3 genes from pre-gastrulation to early somite stages. Mech. Dev..

[12] Kan L., Jalali A., Zhao L.R., Zhou X., McGuire T., Kazanis I., Episkopou V., Bassuk A.G., Kessler J.A. (2007). Dual function of Sox1 in telencephalic progenitor cells. Dev. Biol..

[13] Mitsui K., Tokuzawa Y., Itoh H., Segawa K., Murakami M., Takahashi K., Maruyama M., Maeda M., Yamanaka S. (2003). The homeoprotein Nanog is required for maintenance of pluripotency in mouse epiblast and ES cells. Cell.

[14] Voss A.K., Krebs D.L., Thomas T. (2006). C3G regulates the size of the cerebral cortex neural precursor population. EMBO J..

[15] Kleinman H.K., Martin G.R. (2005). Matrigel: Basement membrane matrix with biological activity. Semin. Cancer Biol..

[16] Hughes C.S., Postovit L.M., Lajoie G.A. (2010). Matrigel: A complex protein mixture required for optimal growth of cell culture. Proteomics.

[17] Choi H.W., Hong Y.J., Kim J.S., Song H., Cho S.G., Bae H., Kim C., Byun S.J., Do J.T. (2017). In vivo differentiation of induced pluripotent stem cells into neural stem cells by chimera formation. PLoS ONE.

[18] Ross H.H., Ambrosio F., Trumbower R.D., Reier P.J., Behrman A.L., Wolf S.L. (2016). Neural stem cell therapy and rehabilitation in the central nervous system: Emerging partnerships. Phys. Ther..

[19] Choi H.W., Joo J.Y., Hong Y.J., Kim J.S., Song H., Lee J.W., Wu G., Schöler H.R., Do J.T. (2016). Distinct enhancer activity of Oct4 in naive and primed mouse pluripotency. Stem Cell Rep..

