# Supplementary material for: Efficient Generation of Neural Stem Cells from Embryonic Stem Cells Using a Three-Dimensional Differentiation System
Source: Int J Mol Sci. 2021 Aug 3;22(15):8322. doi: 10.3390/ijms22158322 (PMC8348082; doi:10.3390/ijms22158322)
Supplement: Supplementary file 1 [file ijms-22-08322-s001.zip › ijms-1313657-SI.pdf]

Supplementary information

Efficient generation of neural stem cells from ES cells through three-dimensional differentiation system

Sang Hoon Yoon[a], Mi Rae Bae[a], Hyeonwoo La, Hyuk Song, Kwonho Hong, and Jeong Tae Do*

Department of Stem Cell and Regenerative Biotechnology, Konkuk Institute of Technology, Konkuk University, 120 Neungdong-ro, Gwangjin-gu, Seoul 05029, Korea

[a] These authors contributed equally to this work.

*Correspondence should be addressed to: J.T. Do

E-mail: dojt@konkuk.ac.kr

Tel.: 82-2-450-3673

Fax: 82-2-455-1044

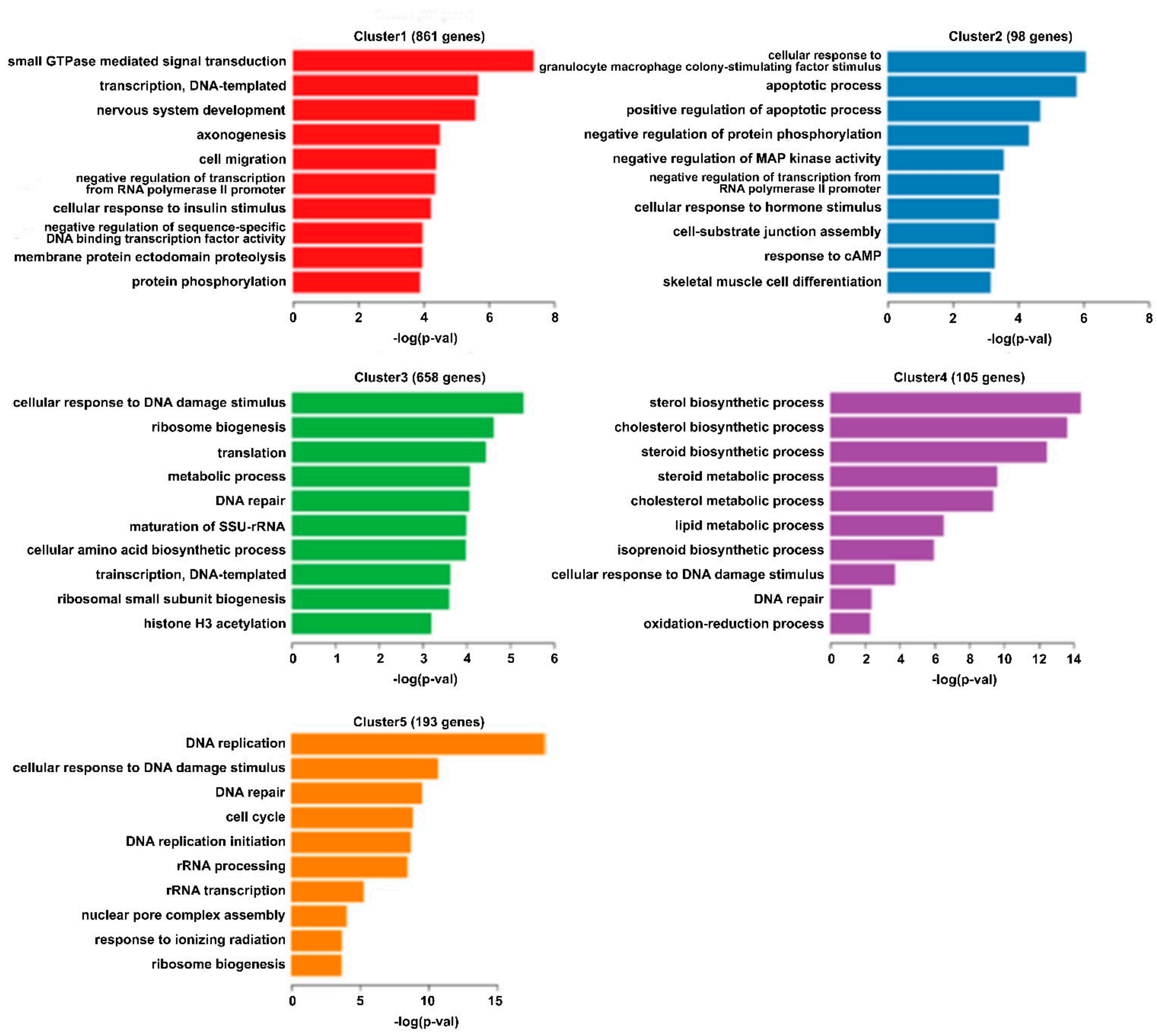

Supplementary Figure S1. Additional Data on Transcriptome Analysis of Established NSCs at the Bulk Levels, Related to Figure 4. DEG clusters analysis of established NSCs, brain-derive NSCs, and Sox-1 GFP ESCs. Q-value, adjusted P-value for multiple comparisons.
